# A simple score for rapid risk assessment of non-high-risk pulmonary embolism

**DOI:** 10.1007/s00392-012-0498-1

**Published:** 2012-08-09

**Authors:** Mareike Lankeit, Dietrich Friesen, Katrin Schäfer, Gerd Hasenfuß, Stavros Konstantinides, Claudia Dellas

**Affiliations:** 1 Department of Cardiology and Pulmonology, Universitätsmedizin Göttingen, Robert-Koch-Strasse 40, 37099 Göttingen, Germany; 2 Department of Cardiology, Democritus University of Thrace, Alexandroupolis, Greece

**Keywords:** Bedside-test, Biomarker, Combination models, Heart-type fatty acid-binding protein (H-FABP), Risk stratification, Pulmonary embolism

## Abstract

**Objective:**

We tested whether bedside testing for H-FABP is, alone or integrated in combination models, useful for rapid risk stratification of non-high-risk PE.

**Methods:**

We prospectively studied 136 normotensive patients with confirmed PE. H-FABP was determined using a qualitative bedside-test showing a positive result for plasma concentration >7 ng/ml.

**Results:**

Overall, 11 patients (8.1 %) had an adverse 30-day outcome. Of 58 patients (42.6 %) with a positive H-FABP bedside-test, 9 (15.5 %) had an unfavourable course compared to 2 of 78 patients (2.6 %) with a negative test result (*p* = 0.009). Logistic regression analysis indicated a sevenfold increased risk for an adverse outcome (95 % CI, 1.45–33.67; *p* = 0.016) for patients with a positive H-FABP bedside-test. Additive prognostic information were obtained by a novel score including the H-FABP bedside-test (1.5 points), tachycardia (2 points), and syncope (1.5 points) (OR 11.57 [2.38–56.24]; *p* = 0.002 for ≥3 points). Increasing points were associated with a continuous exponential increase in the rate of an adverse 30-day outcome (0 % for patients with 0 points and 44.4 % for ≥5 points). Notably, this simple score provided similar prognostic value as the combination of the H-FABP bedside-test with echocardiographic signs of right ventricular dysfunction (OR 12.73 [2.51–64.43]; *p* = 0.002).

**Conclusions:**

Bedside testing for H-FABP appears a useful tool for immediate risk stratification of non-high-risk patients with acute PE, who may be at increased risk of an adverse outcome, in particular if integrated in a novel score without the need of echocardiographic examination.

## Introduction

Acute pulmonary embolism (PE) is a frequent and life-threatening disease. As proposed by current guidelines [1, 2], risk stratification is based on clinical assessment of haemodynamic instability in order to identify patients who are at high risk of early death or life-threatening complications. Furthermore, laboratory biomarkers and imaging procedures can be used for the assessment of right ventricular (RV) dysfunction and injury and thus for classifying non-high-risk patients into an intermediate-risk and a low-risk subgroup. Currently, the most widely used laboratory markers of myocardial (RV) dysfunction and injury, natriuretic peptides [3] and cardiac troponins [4, 5], are characterised by low specificity and positive predictive values and do not, by themselves, justify more aggressive treatment regimens [6]. Therefore, in the past years, strategies for optimising risk stratification of normotensive patients with acute PE have focused on (1) combination models integrating information from laboratory markers and imaging procedures [7–9]; (2) clinical scores of PE severity [10–14]; and (3) novel, promising biomarkers [9, 15].

Recently, we and others demonstrated that heart-type fatty acid-binding protein (H-FABP), an early and sensitive marker of myocardial injury with favourable release kinetics [16], is of prognostic value in patients with acute PE and improves risk stratification of both, unselected [17, 18] and haemodynamic stable patients [19]. In fact, H-FABP appeared superior to cardiac troponins and natriuretic peptides for predicting an adverse outcome [19]. In those studies, H-FABP concentrations were determined using quantitative solid-phase enzyme-linked immunoadsorbent assays based on the sandwich principle (ELISAs) which are currently not available for application in clinical routine. Therefore, a point-of-care test for H-FABP was developed that allows rapid (within 20 min) qualitative determination of H-FABP concentrations in full blood or plasma [20]. This assay emerged as a reliable test for the early diagnosis of acute myocardial infarction [21, 22]. In intermediate-risk patients with acute PE, positive test results indicating H-FABP plasma concentrations above 7 ng/ml were associated with impaired RV function [23].

The aim of the present study was to determine whether bedside testing of H-FABP is—alone or integrated in combination models with other predictors of an adverse outcome—capable of accelerating and simplifying risk stratification of non-high-risk PE.

## Methods

### Patient population and study design

Consecutive patients who were diagnosed with acute symptomatic PE were prospectively studied at the University of Göttingen between October 2005 and April 2009. For inclusion in the study, diagnosis of PE had to be confirmed by an imaging procedure (contrast-enhanced multidetector computed tomography, ventilation–perfusion lung scan, or pulmonary angiography; or by echocardiography showing the presence of mobile thrombi in the right atrium or ventricle, or in the proximal portions of the pulmonary artery) based on the diagnostic algorithms proposed by recent guidelines [1, 2] and those existing before 2008 [24, 25]. Patients were excluded from the study if they met at least one of the following criteria: (1) haemodynamic instability at presentation, defined as the presence of the following: need for cardiopulmonary resuscitation, systolic blood pressure <90 mmHg or drop of systolic blood pressure by ≥40 mmHg for ≥15 min with signs of end-organ hypoperfusion, or need for catecholamine administration to maintain adequate organ perfusion and a systolic blood pressure ≥90 mm Hg; (2) PE being an accidental finding obtained during diagnostic workup for another suspected disease; and (3) denial of consent or withdrawal of previously given consent for participation in the study.

According to the study protocol, and as described previously [15, 19], complete data on baseline clinical, haemodynamic, and laboratory parameters were obtained using a standardised questionnaire. Treatment decisions were made by the physicians caring for the patient according to the mentioned guidelines and not influenced by the study protocol. Study results were not communicated to the clinicians and thus not used to guide the patient’s management or to monitor the effects of treatment during the hospital stay or at any time during the 30-day follow-up period.

A transthoracic echocardiogram was strongly recommended by the study protocol. Right ventricular (RV) dysfunction was defined as dilatation of the right ventricle (end-diastolic diameter >30 mm from the parasternal view, or a right/left ventricle diameter ratio ≥1.0 from the subcostal or apical view) combined with absence of inspiratory collapse of the inferior vena cava, in the absence of left ventricular or mitral valve disease [15, 19, 26].

Thirty-day clinical follow-up data were obtained from all patients included in the study. An adverse 30-day outcome was defined as death from any cause or at least one of the following major complications [15, 19]: (1) need for intravenous catecholamine administration (except for dopamine at a rate of ≤5 μg/kg/min) to maintain adequate tissue perfusion and prevent or treat cardiogenic shock; (2) endotracheal intubation; and (3) cardiopulmonary resuscitation. The causes of death were adjudicated by two of the authors (M.L. and C.D.) by reviewing the patients’ medical records and the results of autopsy if performed.

The study protocol was approved by the Ethical Committee of the University of Göttingen.

### Laboratory parameters and biomarker testing

Venous plasma samples were collected on admission and immediately stored at −80 °C. Samples were later analysed in batches after a single thaw.

Qualitative plasma concentrations of H-FABP were determined using a rapid chromatographic immunoassay (“bedside-test”; CardioDetect^®^ lab, Rennessens GmbH, Berlin, Germany), as previously described [20, 21]. The test, which is approved for plasma, serum and whole blood samples, shows a “positive” test result for H-FABP plasma concentrations above 7 ng/ml, and test results are available within 20 min.

Routine laboratory parameter measurements including the conventional assays for cardiac troponin T (cTnT) and N-terminal pro-brain natriuretic peptide (NT-proBNP) (quantitative electrochemiluminescence immunoassays (Elecsys 1010/2010 analyzer, Roche Diagnostics, Mannheim, Germany) were performed at the Department of Clinical Chemistry of the University of Göttingen. For NT-proBNP, a concentration of 1,000 pg/ml was defined as cut-off value for distinguishing between normal and elevated plasma levels [7, 15], and for cTnT a concentration of 0.03 ng/ml as specified by the manufacturer. The glomerular filtration rate (GFR) was estimated using the Modification of Diet in Renal Disease (MDRD) study equation; renal insufficiency was defined as GFR <60 ml/min/1.73 m^2^ body-surface area.

### Statistical analysis

Continuous variables were found not to follow a normal distribution as tested with the modified Kolmogorov–Smirnov test (Lilliefors test). They were therefore expressed as medians with corresponding 25th and 75th percentiles and compared using the unpaired Mann–Whitney *U* test. Categorical variables were compared using Fisher’s exact test or Chi^2^ test, as appropriate. Receiver operating characteristic (ROC) analysis was used to determine the area under the curve (AUC) of baseline biomarker concentrations and the novel score with regard to an adverse 30-day outcome. Furthermore, ROC analysis was used for defining the optimal cut-off value of the novel score. Sensitivity, specificity, and the positive and negative predictive value of elevated biomarker levels and the novel score were calculated. The prognostic relevance of a positive H-FABP bedside-test, elevated biomarker concentrations, and other baseline parameters (as listed in Table 1) as well as of the combination models with regard to 30-day outcome was estimated using logistic regression analysis. Odds ratios (OR) and the corresponding 95 % confidence intervals (CI) were calculated.

**Table 1:** Baseline characteristics, clinical symptoms, and relevant findings on admission of 136 normotensive (non-high-risk) patients with acute pulmonary embolism

	All study patients (*n* = 136)	Bedside-test negative (*n* = 78)	Bedside-test positive (*n* = 58)	*p* value
Gender (male/female)	56 (41 %)/80 (59 %)	34 (44 %)/44 (56 %)	22 (38 %)/36 (62 %)	0.598
Age (years)	68 (56–76)	65 (48–71)	73 (63–80)	<0.001
Symptoms on admission				
Symptom onset <24 h	82 (60.3 %)	42 (53.8 %)	40 (67.0 %)	0.080
Tachycardia (heart rate ≥100 bpm)	53 (39.0 %)	25 (32.1 %)	28 (48.3 %)	0.075
Dyspnea	110 (80.9 %)	64 (82.1 %)	46 (79.3 %)	0.826
Chest pain	69 (50.7 %)	46 (59.0 %)	23 (39.7 %)	0.037
Syncope	30 (22.1 %)	10 (12.8 %)	20 (34.5 %)	0.003
Comorbidities and risk factors for VTE				
History of DVT or PE	42 (30.1 %)	27 (34.6 %)	15 (25.9 %)	0.349
Immobilisation	49 (36.0 %)	28 (35.9 %)	21 (36.2 %)	1.000
Cancer	26 (19.1 %)	11 (14.1 %)	15 (25.9 %)	0.122
Congestive heart failure	14 (10.3 %)	4 (5.1 %)	10 (17.2 %)	0.042
Coronary artery disease	17 (12.5 %)	8 (10.3 %)	9 (15.5 %)	0.435
Chronic pulmonary disease	20 (14.7 %)	12 (15.4 %)	8 (13.8 %)	1.000
Laboratory parameters and biomarkers				
Creatinine (mg/dl)	0.89 (0.71–1.10)	0.80 (0.70–1.00)	1.00 (0.80–1.30)	<0.001
GFR <60 ml/min/1.73 m^2^	36 (26.5 %)	12 (15.4 %)	24 (41.4 %)	0.001
NT-proBNP (pg/ml)	766 (126–2,371)	309 (82–1,255)	1,605 (343–4,325)	<0.001
NT-proBNP ≥1,000 pg/ml	64 (47.4 %) (*n* = 135)	28 (36.4 %) (*n* = 77)	36 (62.1 %)	0.005
cTnT (ng/ml)	0.01 (0.01–0.05)	0.01 (0.01–0.02)	0.03 (0.01–0.06)	<0.001
cTnT ≥0.03 ng/ml	47 (35.6 %)	19 (24.4 %)	28 (48.3 %)	0.006
RV dysfunction	48 (47.1 %) (*n* = 102)	18 (32.7 %) (*n* = 55)	30 (63.8 %) (*n* = 47)	0.003

Patients were stratified according to a negative or positive H-FABP bedside-test on admission. Data are presented as absolute numbers (percentages) or medians (25th to 75th percentile)

*n* number of patients with available data, *Bpm* beats per minute, *VTE* venous thromboembolism, *DVT* deep vein thrombosis, *PE* pulmonary embolism, *GFR* glomerular filtration rate, *NT-proBNP* N-terminal pro-brain natriuretic peptide, *cTnT* cardiac troponin T, *RV* right ventricular

All tests were two-sided and used a significance level of 0.05. Analyses were performed using the PASW software (version 18.0, Chicago, Illinois, USA).

## Results

### Baseline clinical and laboratory findings

Overall, 136 normotensive patients with acute PE were included in the study (derivation cohort). The baseline clinical characteristics of the study patients are summarised in Table 1. Diagnosis of PE was confirmed by contrast-enhanced multidetector computed tomography (*n* = 120, 88.2 %), ventilation–perfusion lung scan (*n* = 13, 9.6 %), or pulmonary angiography (*n* = 1; 0.7 %). In two patients (1.5 %), the diagnosis of PE was established by echocardiographic criteria (as explained in the “Methods”). Overall, a transthoracic echocardiogram was performed in 102 patients (75.0 %); of these, 48 patients (47.1 %) were diagnosed with RV dysfunction.

On admission, the H-FABP bedside-test was positive in 58 patients (42.6 %). As shown in Table 1, patients with a positive H-FABP bedside-test were older and more frequently diagnosed with congestive heart failure and renal insufficiency. These patients were more likely to present with syncope but less likely to present with chest pain. Cardiac TnT levels ranged from 0.01 to 0.34 ng/ml with a median value of 0.01 (25th to 75th percentile, 0.01–0.05) ng/ml, and 47 patients (35.6 %) had cTnT levels above the cut-off value of 0.03 ng/ml. NT-proBNP levels ranged from 10 to 32,156 pg/ml with a median value of 766 (126–2,371) pg/ml, and 64 patients (47.4 %) had NT-proBNP levels above the cut-off value of 1,000 pg/ml. Both biomarkers were higher in patients with a positive H-FABP bedside-test (*p* < 0.001 each).

### H-FABP bedside testing for predicting early outcome after acute PE

During the acute phase of PE (first 30 days), 11 patients (8.1 %) had an adverse outcome. Overall, 7 patients (5.1 %) died; four deaths were due to PE and three to cancer as the underlying disease. Patients with an adverse 30-day outcome presented more often with a positive H-FABP bedside-test on admission compared to patients with a favourable course (81.8 vs. 39.2 %; *p* = 0.009). As shown in Table 2, a positive H-FABP bedside-test alone was associated with a prognostic sensitivity (82 %) and specificity (61 %). Overall, 15.5 % of the patients with a positive H-FABP bedside-test on admission died or developed life-threatening complications, while 2.6 % of those patients with a negative test had an adverse 30-day outcome. ROC analysis showed an AUC of 0.713 (95 % CI, 0.567–0.859) for the H-FABP bedside-test, compared to 0.654 (0.515–0.792) for cTnT and 0.639 (0.479–0.800) for NT-proBNP.

**Table 2:** Receiver operating characteristics analysis for the predictive value of biomarkers in acute normotensive pulmonary embolism

	Sensitivity	Specificity	PPV	NPV
H-FABP bedside-test positive	0.82	0.61	0.16	0.97
cTnT ≥0.03 ng/ml	0.45	0.66	0.11	0.93
NT-proBNP ≥1,000 pg/ml	0.73	0.55	0.13	0.96

*PPV* positive predictive value, *NPV* negative predictive value, *H-FABP* heart-type fatty acid-binding protein, *cTnT* cardiac troponin T, *NT-proBNP* N-terminal pro-brain natriuretic peptide

Univariable logistic regression analysis indicated a sevenfold increase in the risk of an adverse 30-day outcome (95 % CI, 1.45–33.67; *p* = 0.016) for patients with a positive H-FABP bedside-test. As shown in Table 3, besides a positive H-FABP bedside-test, tachycardia, evidence of RV dysfunction on echocardiography, and syncope were identified as being univariably correlated with a poor outcome, whereas elevation of the established biomarkers cTnT (*p* = 0.432) and NT-proBNP (*p* = 0.094) above their cut-off values did not appear to provide prognostic information.

**Table 3:** Predictors of an adverse 30-day outcome

	OR	95 % CI	*p* value
H-FABP bedside-test positive	6.98	1.45–33.67	0.016
Tachycardia (HR ≥100 bpm)	8.28	1.71–40.04	0.009
RV dysfunction	12.23	1.49–100.58	0.020
Syncope	5.05	1.42–17.94	0.012

Displayed are only variables found to significantly predict an adverse 30-day outcome by univariable analysis. ORs with the respective 95 % CI for an adverse 30-day outcome were calculated by logistic regression analysis

*OR* odds ratio, *CI* confidence interval, *H-FABP* heart-type fatty acid-binding protein, *HR* heart rate, *bpm* beats per minute, *RV* right ventricular

### Integration of the H-FABP bedside-test into combination models

We investigated whether the combination of the H-FABP bedside-test with other predictors of an adverse outcome might further improve risk stratification of acute PE. Indeed, the combination of the H-FABP bedside-test with evidence of RV dysfunction on echocardiography was associated with a 12.73-fold increase in the risk of an adverse 30-day outcome (2.51–64.43; *p* = 0.002), which appeared superior to the prognostic information provided by the H-FABP bedside-test alone (Table 3). Of the 102 patients with an echocardiographic examination on admission, 30 patients (29.4 %) had a positive H-FABP bedside-test and evidence of RV dysfunction on echocardiography and 8 of them (26.7 %) had an adverse 30-day outcome. On the other hand, two of 72 patients (2.8 %) with a negative H-FABP bedside-test and/or a normal echocardiogram died or had serious complications (*p* = 0.001). Overall, the combination model was associated with a prognostic sensitivity of 80 %, a specificity of 76 %, a positive predictive value (PPV) of 27 %, and a negative predictive value (NPV) of 97 %. In comparison, neither cTnT nor NT-proBNP improved the prognostic information provided by echocardiography alone (data not shown).

**Fig. 1:**
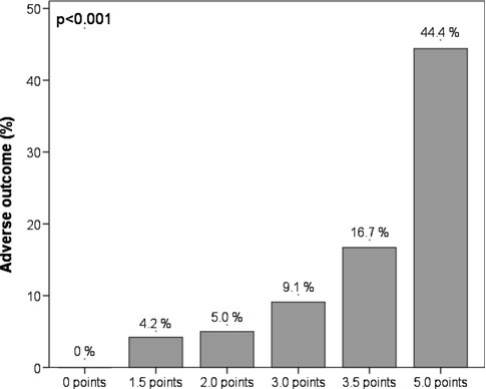
Rate of an adverse 30-day outcome according to the sum of points in the novel score

Since echocardiography may not be available on a round-the-clock basis in many hospitals, and in view of the fact that echocardiographic criteria for the detection of acute RV dysfunction are often vague and poorly standardised [26, 27], we tested whether comparable prognostic information could be obtained by an alternative combination model based on bedside parameters for rapid risk assessment. For this purpose, all clinical variables that were identified as univariable predictors of an adverse 30-day outcome (Table 3) were included in a score and the respective weight was obtained from the regression coefficient of the multivariable logistic regression analysis. Thus, a positive H-FABP bedside-test “weighted” 1.5 points; tachycardia, 2.0 points; and syncope, 1.5 points. By ROC analysis (AUC, 0.847 [0.746–0.949]) we identified an optimal cut-off value of 3.0 points for discriminating between patients with an adverse 30-day outcome and those with a favourable course. Of 44 patients (32.4 %) with a score of ≥3.0 points, 9 patients (20.5 %) developed complications or died during the first 30 days as opposed to only two of 92 patients (2.2 %) with a score of <3 points (*p* = 0.001). The dichotomised score was associated with a sensitivity of 82 %, a specificity of 72 %, a PPV of 20 %, and a NPV of 98 %. Using logistic regression analysis, a score of ≥3.0 points was associated with a nearly 12-fold increase in the risk of an adverse 30-day outcome (OR 11.57 [2.38–56.24]; *p* = 0.002). The prognostic relevance of the novel score remained unaffected if adjusted for age (data not shown). Notably, patients with a score ≥5.0 points had a 13.7-fold increase in the risk of an adverse 30-day outcome ([3.00–62.69]; *p* = 0.001) and a rate of an adverse 30-day outcome of 44.4 % (Fig. 1). Moreover, increasing points in the score were associated with a continuous exponential increase in the rate of an adverse 30-day outcome (Fig. 1; *p* < 0.001 for trend). Importantly, none of the 48 patients with a score of 0 points had an adverse outcome.

## Discussion

Detection of an elevated risk amongst normotensive patients with acute PE remains challenging despite the large number of studies published in the last decade. As currently available biomarker assays share the requirement for time-consuming measurements, the development of bedside-tests for rapid determination of biomarker concentrations appears to be a promising approach. In the present study, we could demonstrate that bedside testing for H-FABP is a useful tool for immediate risk stratification of normotensive patients with acute PE. Besides the association of a positive test result with an adverse early outcome, the H-FABP bedside-test appeared to provide valuable prognostic information when integrated into a novel, “simple” clinical score also including tachycardia and syncope.

H-FABP is a promising biomarker of myocardial injury with favourable release kinetics [16]. Due to its small molecular size (15 kDa) and its cytoplasmatic location, H-FABP plasma concentrations rise as early as 30 min after the onset of myocardial ischemia, peak at 6–8 h, and return to normal within 24–30 h [28]. We and others previously demonstrated that H-FABP is of prognostic value in patients with acute PE and improves risk stratification of both unselected and normotensive patients [17–19]. However, widespread clinical use of H-FABP has been prevented by the need for a time-consuming solid-phase ELISA. A novel point-of-care test, the CardioDetect^®^ lab assay, allows qualitative determination of H-FABP concentrations (positive vs. negative test result) within 20 min [20]. We could now demonstrate in 136 normotensive patients with acute PE that the H-FABP bedside-test emerged, besides tachycardia, evidence of RV dysfunction on echocardiography, and syncope, as predictor of an adverse early outcome.

Multimarker models integrating information obtained from echocardiography (evidence or exclusion of RV dysfunction) in combination with laboratory biomarkers (such as growth-differentiation factor-15 (GDF-15) [9], NT-proBNP [7], cTnT [8, 29]/hsTnT [15], or H-FABP [18, 19]) have been reported to improve risk stratification of acute PE. This could be confirmed in the present study, in which the combination of echocardiographic evidence of RV dysfunction with the H-FABP bedside-test predicted a 12.7-fold increase in the risk of an adverse 30-day outcome. However, it needs to be kept in mind that echocardiographic criteria for defining acute RV dysfunction are poorly standardised and may vary widely between hospitals, ultrasound laboratories, and examiners [26, 30]. Moreover, echocardiography may not be available outside the working hours, especially in smaller community hospitals. This problem might be expected to occur even more frequently in normotensive patients with acute PE, who are generally not considered “critically ill”. Therefore, we developed a “simple” clinical score derived from the significant predictors of an adverse 30-day outcome at univariable logistic regression (excluding echocardiographic information on RV function). This score included the H-FABP bedside-test (a positive result was assigned 1.5 points), tachycardia (heart rate above 100 beats/min on admission assigned 2 points), and syncope (presence of syncope assigned 1.5 points). The present score thus integrates weighed prognostic information from baseline clinical parameters which are easy to determine in clinical routine, in combination with a biomarker which can be measured by a point-of-care test within 20 min. We found that patients with a score above the calculated cut-off value of 3.0 points had a 12-fold increase in the risk of an adverse 30-day outcome. In fact, this was nearly identical with the prognostic information provided by the combination of echocardiography and the H-FABP bedside-test. Importantly, none of the 48 patients (35.3 %) with a score of 0 had an adverse outcome, while two (2.8 %) of the 72 patients with a negative H-FABP bedside-test and/or normal echocardiogram had an unfavourable course. Further, an increasing score was associated with a continuous exponential increase in the rate of an adverse 30-day outcome (Fig. 1). Therefore, calculation of the new score may offer a valuable and fast alternative for immediate risk assessment of normotensive patients with acute PE if echocardiography is not available. Pending external validation of our results, the novel score might simplify and accelerate risk stratification of PE patients in clinical routine in the future.

In the last years, a number of clinical prediction rules were developed for prognostic assessment of patients with acute PE. The pulmonary embolism severity index (PESI) [10] focuses on 11 different weighted patient characteristics such as comorbidities and baseline clinical parameters and allows stratification into five severity classes. Its simplified version (sPESI) [11] reduces the technical complexity of the original prediction rule by focusing on six equally weighted variables. However, the (s)PESI, as well as a score developed by Uresandi et al. [13], appears to be more suitable for the identification of low-risk patients than for patients with an elevated risk of an adverse outcome. The Geneva Score [14] focuses on six different variables such as comorbidities and haemodynamic parameters but also needs an ultrasound examination of the leg veins which may not be available in many hospitals. The PREP score [31] is based on five different weighted clinical, echocardiographic [RV/LV ratio), and biochemical variables (brain natriuretic peptide (BNP)] and allows stratification in five severity classes. Another recently proposed prognostic model consists of NT-proBNP, D-dimer concentrations, heart rate, and cancer with a total score range from 0 to 37 points [12]. The novel score which we developed in the present study is the first to integrate baseline clinical parameters and prognostic information obtained from a cardiac biomarker (in contrast to the (s)PESI which does not account for right ventricular dysfunction) without the need of a transthoracic echocardiogram (as in the PREP score) or ultrasound examination of the leg veins (as in the Geneva Score). Our score is characterised by low complexity, with only three differently weighed variables, and by the use of a bedside-test for fast and immediate risk stratification.

As the purpose of our study was to investigate the performance of the H-FABP bedside-test, we did not compare it with the quantitative solid-phase enzyme-linked immunoadsorbent assay (ELISA) for determination of H-FABP used in previous studies. Hence, our results may be limited regarding the universal use of H-FABP values in the novel score. However, findings from Boscheri et al. [23] indicate that the CardioDetect^®^ lab assay performs as reliably as the H-FABP ELISA (HyCult biotechnology b.v., Uden, Netherlands). Another limitation is the missing of an external validation cohort. Thus, the novel score and the application of the bedside-test require external validation in normotensive patients with acute PE and we are hereby providing the base of further clinical studies. Moreover, the relatively small number of events in the acute phase of normotensive PE is a potential limitation of the present study.

In conclusion, we could demonstrate that the H-FABP bedside-test reliably identified an increased risk of an adverse early outcome in a derivation cohort of 136 normotensive patients with acute PE. Importantly, the integration of the bedside-test into a novel score also including two baseline clinical parameters, namely tachycardia and syncope, may offer a simple, readily available and fast approach to immediate risk assessment of PE, particularly if imaging of the right ventricle cannot be rapidly obtained.
